# Fungal colonization in patients with chronic respiratory diseases from Himalayan region of India

**DOI:** 10.1186/1476-0711-9-28

**Published:** 2010-09-20

**Authors:** Debasis Biswas, Sonal Agarwal, Girish Sindhwani, Jagdish Rawat

**Affiliations:** 1 Deptartment of Microbiology, Himalayan Institute of Medical Sciences, Swami Ram Nagar, Jolly Grant, Dehradun, Uttarakhand, 248140, India; 2 Department of Pulmonary Medicine, Himalayan Institute of Medical Sciences, Swami Ram Nagar, Jolly Grant, Dehradun, Uttarakhand, 248140, India

## Abstract

**Background:**

We screened patients with chronic respiratory diseases for microbiological and serological evidences of fungal colonisation; in order to determine its prevalence in this group of patients, examine potential clinical and radiological predictors of fungal colonisation and characterise fungal agents associated with individual diseases.

**Methods:**

BAL samples from 60 consecutive patients were subjected to microscopy and culture for fungal agents. Serum samples were analysed for precipitin antibodies to Aspergillus antigen and Candida cytoplasmic antigen. Statistical significance in the difference of fungal recovery between patient groups was determined using the Chi-square test.

**Results:**

The major diagnostic groups included patients with bronchogenic carcinoma (n = 31) and tubercular sequelae (n = 16). In all, 28 patients (46.7%) were culture-positive, with Candida and Aspergillus being recovered from 14 and 13 patients respectively. Twenty-one patients (35%) showed presence of precipitin antibodies. Patients with bronchogenic carcinoma showed increased predilection for colonisation with Aspergillus, while Candida was recovered more commonly in tubercular sequelae (p = 0.02). There was no statistically significant association between culture-positivity and specific risk factors/radiological findings.

**Conclusion:**

The point-prevalence of fungal colonization was almost 50%. The combination of fungal culture and serology helped improve diagnostic sensitivity. An interesting predilection was observed for Aspergillus and Candida, to preferentially infect patients with Bronchogenic carcinoma and Tubercular sequelae respectively. In absence of specific predictors, the possibility of fungal colonization needs to be explored actively in these patients.

## Background

Fungal infections have emerged as a world-wide health care problem in recent years [1], owing to the extensive use of broad-spectrum antibiotics [2], long-term use of immunosuppressive agents, increasing use of hyperalimentation and indwelling devices [3] and the increasing population of terminally ill, debilitated and immunocompromised patients [4]. In tune with this general trend, there has been a phenomenal rise in the occurrence of fungal lung infections over the last two decades [5], a significant fraction of which is community-acquired [1]. Specific diagnosis of fungal pneumonia assumes importance in view of the different therapeutic strategies involved and the higher mortality associated with acute invasive fungal infection. Unfortunately, diagnosis of fungal respiratory infection is always difficult, owing to the lack of pathognomonic clinical features, contamination of the non-invasive samples like sputum with normal commensal flora and difficulty in obtaining invasive samples like translaryngeal aspirate and lung biopsy. A previous study has shown that *Candida *colonization could be found in the respiratory samples obtained by Bronchoalveolar lavage (BAL), endotracheal aspirate or protected specimen brushing in critically ill patients [6]. Since patients with chronic lung pathology provide a suitable nidus for fungal colonization, we hypothesized that screening such patients for fungal colonization of the respiratory tract would enable us to identify individuals requiring closer monitoring for the development of possible complications like acute invasive fungal infection or dissemination via hematogenous spread. The present study was designed with the purpose of characterizing the fungal pathogens associated with particular pulmonary diseases, and correlating between the individual fungal pathogens and the profile of risk factors, radiological presentations and clinical conditions in these patients.

## Methods

### Recruitment of patients and collection of clinical samples

The study extended over a period of 12 months and included 60 consecutive patients with chronic lung diseases who required bronchoscopic examination for diagnostic purposes. Patients suffering from active Tuberculosis, pneumonitis and HIV-positive patients were excluded. The study protocol was duly approved by the institutional research and ethics committees. Signed informed consent was obtained from all the study participants.

Bronchoalveolar lavage (BAL) sample was collected aseptically with the help of flexible bronchoscope (Olympus Corporation; BF type TE2), attached to a light source (Olympus CLK-4) and digital signal processing camera. The selection of the site for collection of BAL fluid was guided by prior imaging studies and local inspection of the disease site. As advocated by Jourdain *et al *no endobronchial suction was attempted during the advancement of the bronchoscope to avoid contamination with upper airway flora and the first two aliquots were discarded [7]. The BAL fluid was collected in a sterile container and transported immediately to the mycology laboratory. None of the patients had received antibiotics for at least 2 weeks prior to the collection of BAL fluid. Serum samples were also collected from the same patients and stored at -20°C for serological analysis.

### Mycological processing

Centrifuged deposits prepared from BAL samples were used for direct microscopy and culture on Sabouraud's dextrose agar (SDA) with and without antibiotics and Czapek Dox Agar. The clinical significance of these isolates were determined by ensuring that contamination with oral flora had not taken place during sampling and by correlating the culture record with observation made during direct microscopy. Moreover, the growth was considered to be significant only when the same fungal agent was isolated from the different culture tubes.

The significant fungal isolates recovered on culture were identified to the species level, using standard mycological procedures. For *Candida sp*. isolates, the tests performed included germ tube test, Dalmau culture plate, sugar assimilation and fermentation tests and ChromAgar. Identification of *Aspergillus sp*. was done by examining the colony morphology and microscopic morphology of wet mounts prepared from the fungal growth, before and after slide culture examination. *Cryptococcus neoformans *was identified by characteristic colony morphology, India ink preparation

### Immunodiffusion test with Candida and Aspergillus antigens

For immunodiffusion tests, Aspergillus antigen and antisera were commercially procured (Adaltis Inc, Italy) and Candida cytoplasmic antigen and corresponding antisera were kindly provided by Prof. Arunaloke Chakrabarti, Deptt of Medical Microbiology, Post Graduate Institute of Medical Education & Research, Chandigarh, India. Double diffusion test was done at 25°C by Oudin-Ouchterlony's agar gel diffusion method and the immunodiffusion plates were observed for a week to detect the presence of precipitin bands. On appearance, the precipitin bands were stained in amido black solution.

### Statistical analysis

The Chi-square test was used to examine if there was statistically significant difference in the fungal recovery rates between patients belonging to different diagnostic categories and having different co-morbid conditions or risk factors. Chi-square test was also used to analyse possible association between fungal recovery and specific radiological findings in Chest X-ray. Yates' corrected Chi-square test was employed to analyse differences in the nature of fungal isolates recovered from patients with Bronchogenic carcinoma and tubercular sequelae.

## Results

### Clinical profile of patients

The study group comprised of a total of 60 patients (44 males) with ages ranging from 40 to 85 years (median age being 55 years). Thirty one patients (51.7%) presented with bronchogenic carcinoma, while 16 patients (26.7%) had tubercular sequelae like persisting cavity, collapse, fibrosis, etc. Among the different co-morbid conditions and risk factors, the common associations included diabetes mellitus (DM), severe anaemia (Hb <9%), use of steroids, alcoholism and chronic carrier state of HBV. While 10 patients had normal chest X-ray, radiological abnormalities were detected in 50 patients, with consolidation (n = 10), collapse (n = 9) and nodular lesion (n = 8) being the common findings.

### Recovery of fungal agents

Direct microscopic examination revealed the presence of septate hyphae with dichotomous branching in 10 patients and budding yeast cells in 11 patients. Fungal culture yielded *Aspergillus sp*. in 13 patients (including *A. flavus *in 6 patients, *A. fumigatus *in 4 patients and *A. niger *in 3 patients) and *Candida sp*. in 14 (*C. albicans *and *C. tropicalis *being isolated from 12 and 2 patients respectively). One patient, with chronic interstitial lung disease, showed growth of *Cryptococcus neoformans*. The distribution of the fungal isolates with respect to the diagnostic groups is represented in Table 1. In all, 28 patients (46.7%) were culture-positive.

**Table 1:** Recovery of fungal agents with respect to clinical diagnosis

*Clinical Diagnosis*	*No. of patients*	*Culture +ve patients (%)*	*Isolates*	*No. of Isolates (%)*
Bronchogenic carcinoma	31	10 (32.3)	A. flavus	5 (16.1)
			
			C. albicans	2 (6.4)
			
			A. niger	2 (6.4)
			
			A. fumigatus	1 (3.2)

Tubercular sequelae	16	7 (43.8)	C. albicans	5 (31.3)
			
			C. tropicalis	1 (6.3)
			
			A. fumigatus	1 (6.3)

Bronchiectasis	5	5 (100)	C. albicans	3(60)
			
			A. flavus	1 (20)
			
			A. fumigatus	1 (20)

Pleural effusion	2	1 (50)	A. niger	1 (50)

Bronchial asthma	2	2 (100)	C. albicans	1 (50)
			
			A. fumigatus	1 (50)

COPD	3	2 (66.7)	C. albicans	1 (33.3)
			
			C. tropicalis	1 (33.3)

Interstitial lung disease	1	1(100)	C. neoformans	1(100)

### Detection of anti-fungal precipitin antibodies

Twenty one of the recruited patients (35%) reacted positively in the immunodiffusion assays. Of the 13 and 14 patients who showed growth of *Aspergillus sp*. and *Candida sp*. in their BAL samples, 7 (53.8%) and 8 (57.1%) patients respectively were positive for the corresponding anti-fungal antibodies in the immunodiffusion assay. Similarly among the serum samples from 47 and 46 culture-negative patients, 45 (95.7%) and 42 (91.3%) patients respectively were found to be negative for precipitin antibodies. Hence, taking culture as the gold standard, immunodiffusion had a sensitivity of 53.8% and a specificity of 95.7%. The positive and negative predictive values of immunodiffusion assay were 77.7% and 88.2% respectively. The corresponding figures for detection of anti-Candida antibodies were 57.1%, 91.3%, 66.7% and 87.5% respectively.

### Correlation of microbiological and serological evidences of fungal infection

In all, 15 of the 27 culture-positive patients (55.5%) were also positive in immunodiffusion tests and culture-positivity, in absence of precipitin antibodies, was observed in 12 patients. Eight of these 12 patients had co-morbid immunosuppressive factors, like DM (3 patients), cancer chemotherapy (2 patients), steroid use (1 patient), chronic renal failure (1 patient) and chronic carriage of HBV (I patient). Six patients were positive for precipitin bands, despite being culture-negative. Interestingly, 5 of these 6 patients had bronchogenic carcinoma, while the sixth patient had pleural effusion of unresolved aetiology (Table 2).

**Table 2:** Results of Immunodiffusion assay for fungus-specific antibodies

	Results of Immnodiffusion assay	Culture positive	Culture negative	Total
Aspergillus-specific antibodies	Immunodiffusion positive	7	2	9
	
	Immunodiffusion negative	6	45	51
	
	Total	13	47	60

Candida-specific Antibodies	Immunodiffusion positive	8	4	12
	
	Immunodiffusion negative	6	42	48
	
	Total	14	46	60

### Clinical and radiological associates of fungal infection

We next analysed whether the incidence of fungal colonization in the recruited patients had any association with their clinical condition, presence of specific risk factors or radiological findings. Though the difference in culture-positivity rates between patients with Bronchogenic carcinoma and tubercular sequelae was statistically non-significant (p = 0.4), the nature of fungal isolates were different between the two clinical conditions. Among patients with Bronchogenic carcinoma, 8 patients showed growth of *Aspergillus sp*. while *Candida sp*. was recovered from 2. In contrast, among patients with tubercular sequelae, *Candida sp*. was recovered from 6 patients while only a single patient showed growth of *Aspergillus sp*. This difference in the nature of fungal pathogens recovered from patients with Bronchogenic carcinoma and tubercular sequelae was statistically significant (p = 0.02; Yates' corrected Chi-square test).

Diabetes mellitus was found to be the commonest risk factor associated with the isolation of *Candida albicans*. Out of 12 patients having C*andida albicans *as a fungal isolate, 5 were having diabetes mellitus as a predisposing factor (Table 3). However, there was no statistically significant association between culture-positivity and presence of any risk factor (p = 0.5). There was no difference, as well, in the fungal recovery rates between male and female patients. Similarly, none of the radiological patterns showed significantly higher association with fungal recovery (p = 0.7). Interestingly, even normal chest radiography was associated with fungal growth in 5 of the 10 patients. The growth of *Cryptococcus neoformans *was associated with reticulo-nodular lesion on chest radiography (Table 4).

**Table 3:** Recovery of fungal agents with respect to different risk factors

*Co-morbid conditions/risk factors*	*No. of patients*	*Culture +ve patients (%)*	*Isolates*	*No. of Isolates (%)*
Diabetes mellitus	7	5 (71.4)	C. albicans	5 (71.4)

Steroid use	3	2 (66. 7)	C. albicans	1 (33.3)
			
			A. flavus	1 (33.3)

Chronic carrier HBV	3	2 (66. 7)	A. flavus	1 (33.3)
			
			A. fumigatus	1 (33.3)

Post renal transplant	1	1 (100)	C. albicans	1 (100)

Pancytopenia	1	1 (100)	C. albicans	1 (100)

Alcoholism	3	1 (33.3)	C. albicans	1 (100)

Anaemia (Hb < 9%)	6	3 (50)	C. albicans	2 (33.3)
			
			A. niger	1 (16.7)

CRF	1	1 (100)	C. albicans	1 (100)

Cirrhosis	1	1 (100)	C. albicans	1 (100)

**Table 4:** Association of different fungal isolates with radiological findings

*Radiological finding*	*No. of patients*	*Culture +ve patients (%)*	*Isolates*	*No. of Isolates (%)*
Consolidation	18	9 (50)	C. albicans	5(27.8)
			
			A. flavus	2(11.1)
			
			A. fumigatus	2(11.1)

Collapse	9	4 (44.4)	A. flavus	2(22.2)
			
			A. fumigatus	1(11.1)
			
			C. tropicalis	1(11.1)

Nodular lesion	8	2 (25)	C. albicans	1(12.5)
			
			A. niger	1(12.5)

Consolidation + Pleural effusion	5	3 (60)	C. tropicalis	1(20)
			
			A. flavus	1(20)
			
			A. fumigatus	1(20)

Normal	10	5 (50)	C. albicans	3(30)
			
			A. flavus	1(10)
			
			A. niger	1(10)

Pleural effusion	2	1 (50)	A. niger	1(50)

Fibrotic change + Pleural thickness	3	1 (33.3)	C. albicans	1(33.3)

Cavity	4	2 (50)	C. albicans	2(50)

Reticulonodular lesion	1	1 (100)	C. neoformans	1(100)

## Discussion

The literature is replete with reports of fungal lung infections occurring in the background of overtly immunocompromising conditions like transplantation, haematological malignancy, neutropenia and HIV/AIDS. Though several authors have examined the incidence of infection with specific fungal agents in the context of chronic lung pathology, studies on pulmonary mycoses, as a group, have been relatively limited. In this study we report the profile of fungal colonization of the respiratory tract in patients presenting with different chronic respiratory conditions in a tertiary care teaching hospital located in the Himalayan region of India. We observe that 46.7% of the patients recruited in our study were culture-positive for fungal agents and 35% demonstrated presence of anti-fungal precipitin antibodies. Fungal colonization was irrespective of the underlying lung pathology and could not be predicted by the presence of any specific co-morbid condition, risk factor or radiological presentation. Moreover, a normal chest X-ray could not be used to exclude the possibility of accompanying pulmonary fungal infection. Interestingly, the identity of the fungi recovered was different between the major diagnostic categories with significant predilection being observed for colonisation with *Aspergillus sp*. in patients suffering from Bronchogenic carcinoma and for *Candida sp*. among patients with Tubercular sequelae.

Though it is difficult to make a confirmed distinction between fungal colonisation of the lung and active fungal infection [1], we considered our patients to be colonised since none of our patients satisfied the criteria for "proven", "probable" or "possible" Invasive Fungal Disease, as proposed in the revised definitions of invasive fungal disease from the European Organization for Research and Treatment of Cancer/Invasive Fungal Infections Cooperative Group and the National Institute of Allergy and Infectious Diseases Mycoses Study Group (EORTC/MSG) Consensus Group [8]. None of these patients showed any radiological abnormality which could not be explained by their underlying lung pathology. Further follow-up study on patient progress is required to determine whether such colonization is a prelude to the development of co-morbidity due to invasive or disseminated fungal disease and if so, to work out the time kinetics and nature of such progression. However, none of our patients developed invasive fungal infection till the completion of the study.

Though *Candida sp*. and *Aspergillus sp*. constitute the bulk of fungi reported in pulmonary mycoses, their relative proportion and species distribution have shown considerable geographical variation. In the present study, *Candida albicans *was the most frequent isolate, being recovered from 42.9% of patients, followed by *Aspergillus flavus *(21.4%), *Aspergillus fumigatus *(14.3%), *Aspergillus niger *(10.7%), *Candida tropicalis *(7.1%) and *Cryptococcus neoformans *(3.6%). In a similar study on Candida infection in chronic pulmonary conditions, Phukan *et al*. reported an isolation rate of 50% from sputum specimens, with *C. albicans *and *C. tropicalis *predominating [9]. In studies on pulmonary Aspergillosis occurring in chronic lung diseases, Kurhade *et al *[10] and Shahid *et al *[11] have reported isolation of the fungus from 16.3% and 14.7% of cases of chronic respiratory diseases, using sputum and BAL samples respectively. The species distribution of Aspergillus showed some difference between these studies and the present study. *Aspergillus fumigatus *accounted for 80% of the isolates in the former study and Shahid *et al *[11] reported *Aspergillus fumigatus *as the predominant species being isolated from 69.2% cases. Our findings are consistent with reports from other centres in India, in which isolation rate of *A. flavus *largely exceeded that of *A. fumigatus*, underscoring the importance of geographical differences in fungal recovery [12].

The performance of serological tests in the diagnosis of pulmonary mycoses is dependent on a number of factors including the choices of antigen and assay and the immune status of patients. Discordant result between culture and immunodiffusion assays was observed by us in 18 of the 60 (30%) patients. Among them, immunocompromising conditions were observed in 8 of the 12 patients who were culture-positive but immunodiffusion-negative. In view of the ubiquity of common fungi, serological evidence has been reported to assist in confirming fungal infection and add to the clinical significance of fungal recovery [13]. However, interpretation of serological assays becomes difficult in the presence of immunocompromised clinical status [10,14] and invasive fungal infections [15]. In our study, 5 of the 6 patients who were immunodiffusion-positive but culture-negative had bronchogenic carcinoma. The sixth patient had pleural effusion of unresolved aetiology, in whom the possibility of bronchogenic carcinoma could not be excluded. Fungal recovery may be difficult in bronchogenic carcicoma because of non-communication of the lesion with main bronchus [11] and might have been improved in our study on including multiple specimens. Similar observations have been reported by Malik et al who found serology to be more sensitive than culture for the detection of Aspergillosis in patients with bronchogenic carcinoma [16]. We observed seropositivity for Aspergillus antigen in 15% of recruited patients, which is in broad agreement with figures reported in similar studies [10,11,13,17,18].

Owing to the presence of pre-existing respiratory pathology in the recruited patients, it is difficult to establish a causal relationship between the culture-positivity and radiological abnormality. However, 50 of our patients had radiological abnormalities in their chest X-ray, of whom 23 patients were culture-positive for fungal agents. On the other hand, 5 of our culture-positive patients had normal Chest X-ray. Bronchoscopic examnation of all of these 5 patients revealed the presence of central tumors, which could account for the normal appearance on chest X-ray.

In the present study, we report for the first time, serological evidence of Candidiasis in patients with chronic respiratory diseases. Immunodiffusion test with cytoplasmic antigen of *Candida albicans *was positive in 12 of the 60 patients (20%). Of them, 8 patients were culture-positive for Candida and the remaining 4 patients were culture-negative. Sero-reactivity to the 47 kD cytoplasmic antigen has been shown in previous studies to discriminate between patients with deep-seated invasive Candidiasis and superficial Candidiasis and has been proved to be a valuable adjunct in the diagnosis of invasive candidiasis [19,20]. In view of these findings, we used the cytoplasmic antigen of *Candida albicans *in an immunodiffusion format and detected precipitin antibodies in 57.1% of our patients.

The point prevalence of fungal colonization was found to be almost 50% in our patients. Since no association was observed with clinical diagnosis, co-existing risk factors and radiological findings, it is imperative that the possibility of accompanying fungal colonization should be explored meticulously in such patients. An interesting predilection was observed for *Aspergillus sp*. and *Candida sp*. to preferentially infect patients with Bronchoenic carcinoma and Tubercular sequelae respectively. Till further evidence is generated to establish the prognostic implications of fungal colonization with certainty, these patients should be closely monitored, in order to enable earlier detection of invasive or disseminated fungal infection.

## Competing interests

The authors declare that they have no competing interests.

## Authors' contributions

DB designed the study, supervised the laboratory experiments, analysed the data and drafted the manuscript. SA was involved in performing the laboratory experiments and in data collection and analysis. GS and JR performed the clinical examination and recruitment of the patients, data collection and analysis. All authors have critically reviewed the manuscript, approved of its contents and consented to its communication for publication.
